# Site-specific CRISPR-based mitochondrial DNA manipulation is limited by gRNA import

**DOI:** 10.1038/s41598-022-21794-0

**Published:** 2022-11-04

**Authors:** Ludwig Schmiderer, David Yudovich, Leal Oburoglu, Martin Hjort, Jonas Larsson

**Affiliations:** 1 Division of Molecular Medicine and Gene Therapy, Department of Laboratory Medicine and Lund Stem Cell Center, Lund University, 221 00 Lund, Sweden; 2 Chemical Biology and Therapeutics, Department of Experimental Medical Science, Lund University, 221 00 Lund, Sweden; 3 MBC Biolabs, Navan Technologies, San Carlos, CA 94070 USA; 4 BMC A12, Lund University, 221 84 Lund, Sweden

**Keywords:** Molecular biology, Biological techniques, Genetic transduction, Genetic vectors

## Abstract

Achieving CRISPR Cas9-based manipulation of mitochondrial DNA (mtDNA) has been a long-standing goal and would be of great relevance for disease modeling and for clinical applications. In this project, we aimed to deliver Cas9 into the mitochondria of human cells and analyzed Cas9-induced mtDNA cleavage and measured the resulting mtDNA depletion with multiplexed qPCR. In initial experiments, we found that measuring subtle effects on mtDNA copy numbers is challenging because of high biological variability, and detected no significant Cas9-caused mtDNA degradation. To overcome the challenge of being able to detect Cas9 activity on mtDNA, we delivered cytosine base editor Cas9-BE3 to mitochondria and measured its effect (C →  T mutations) on mtDNA. Unlike regular Cas9-cutting, this leaves a permanent mark on mtDNA that can be detected with amplicon sequencing, even if the efficiency is low. We detected low levels of C → T mutations in cells that were exposed to mitochondrially targeted Cas9-BE3, but, surprisingly, these occurred regardless of whether a guide RNA (gRNA) specific to the targeted site, or non-targeting gRNA was used. This unspecific off-target activity shows that Cas9-BE3 can technically edit mtDNA, but also strongly indicates that gRNA import to mitochondria was not successful. Going forward mitochondria-targeted Cas9 base editors will be a useful tool for validating successful gRNA delivery to mitochondria without the ambiguity of approaches that rely on quantifying mtDNA copy numbers.

## Introduction

Mitochondria are the primary site of energy production in the cell and play a central role in a variety of cellular processes. Unlike other organelles, mitochondria carry their own DNA, which is circular and 16.6 kb long, and maternally inherited. Most cell types contain hundreds of mitochondrial DNA (mtDNA) copies, which encode 13 essential subunits of the electron transport chain and ATP synthase, 22 tRNAs, and 2 rRNAs that are required for protein synthesis within mitochondria^1^.

Mutations and deletions within the mitochondrial genome can cause devastating multi-system disorders which cannot be cured with currently approved treatments^2^. Mutated mtDNA can impair the ability of cells to produce energy efficiently, and tissues with high energy demand (e.g. muscles and brain) are particularly impacted by these mutations^3^. Common disease phenotypes include neurological conditions, muscle weakness, deafness, blindness, failure to thrive, and early death^4^. In many mtDNA-related diseases, mutated and regular wild-type mtDNA (wt-mtDNA) coexist within the same cell in a heteroplasmic state^5,6^. The severity of the cellular dysfunction and the resulting diseases is dependent on the ratio between mutated and wt-mtDNA^6,7^.

It has been shown that cellular dysfunction can be ameliorated by selectively eliminating mutant mtDNA. This can be achieved by using site-specific endonucleases that can recognize and cut the sequence of mutant mtDNA, while leaving wt-mtDNA unaffected. Rapid degradation of linearized mtDNA is facilitated by components of the mtDNA replication machinery^8^. wt-mtDNA then repopulates the mitochondria, and normal oxidative phosphorylation is restored. There are hundreds of different disease-causing mtDNA mutations. Previous studies focused on the use of TALENs^9–11^, ZFNs^12–15^, or meganucleases^16^ to target some of these mutations and deletions and successfully eliminated mutant mtDNA in heteroplasmic cell lines and animals. Though these restriction enzymes can be programmed to target specific sequences, for every new DNA target sequence the protein itself has to be redesigned and validated. Protein engineering and functional validation is a complicated and slow process. The CRISPR Cas9 system is a more flexible alternative to the previously used endonucleases. Therefore, we attempted to adapt the CRISPR-Cas9 system to work in mitochondria, in order to extend its benefits from nuclear DNA to mtDNA.

## Results and discussion

CRISPR Cas9 is a two component ribonucleoprotein (RNP) that cuts at a site that is defined by a guide RNA (gRNA) component^17–19^. To achieve specific cutting of mitochondrial DNA, both the Cas9 protein and gRNA must be targeted to the mitochondrial matrix. Protein import to mitochondria can be facilitated by attaching a mitochondrial localization sequence (MLS) to the N-terminal end of a peptide chain^20^. The efficiency of this process can be improved by also attaching a 3’-UTR, which can facilitate mRNA transport to, and translation at mitochondria-bound ribosomes^21,22^.

**Figure 1:**
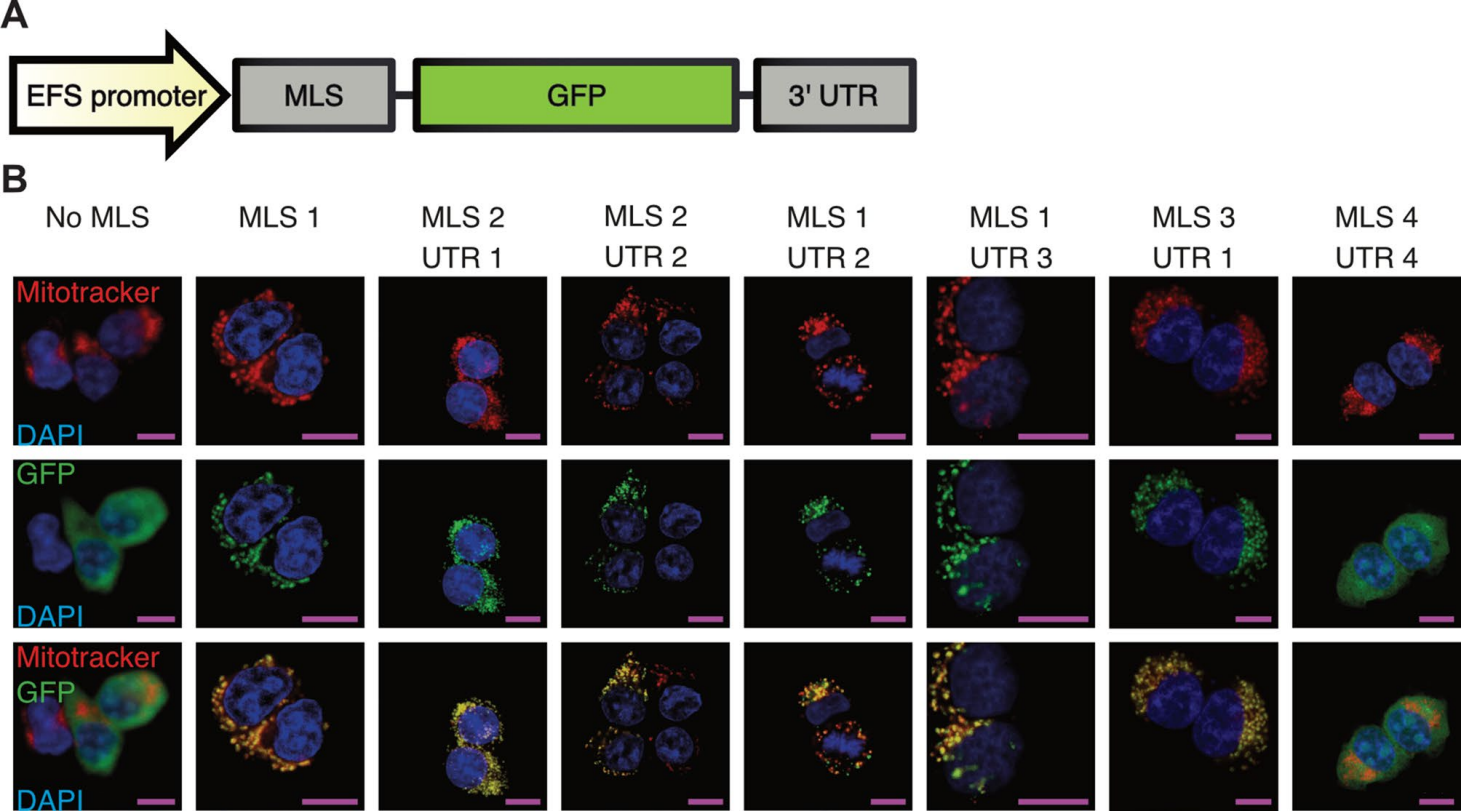
Import of GFP into mitochondria of HEK 293T cells. (**A**) Different MLS/UTR combinations were attached to GFP to facilitate import into mitochondria. (**B**) Representative images showing HEK 293T cells transfected with different constructs. GFP is shown in green, DAPI in blue, and Mitotracker CMXRos in red. Most MLS/UTR combinations lead to clear colocalization of GFP and mitochondria. Scale bars: 10 μm.

**Figure 2:**
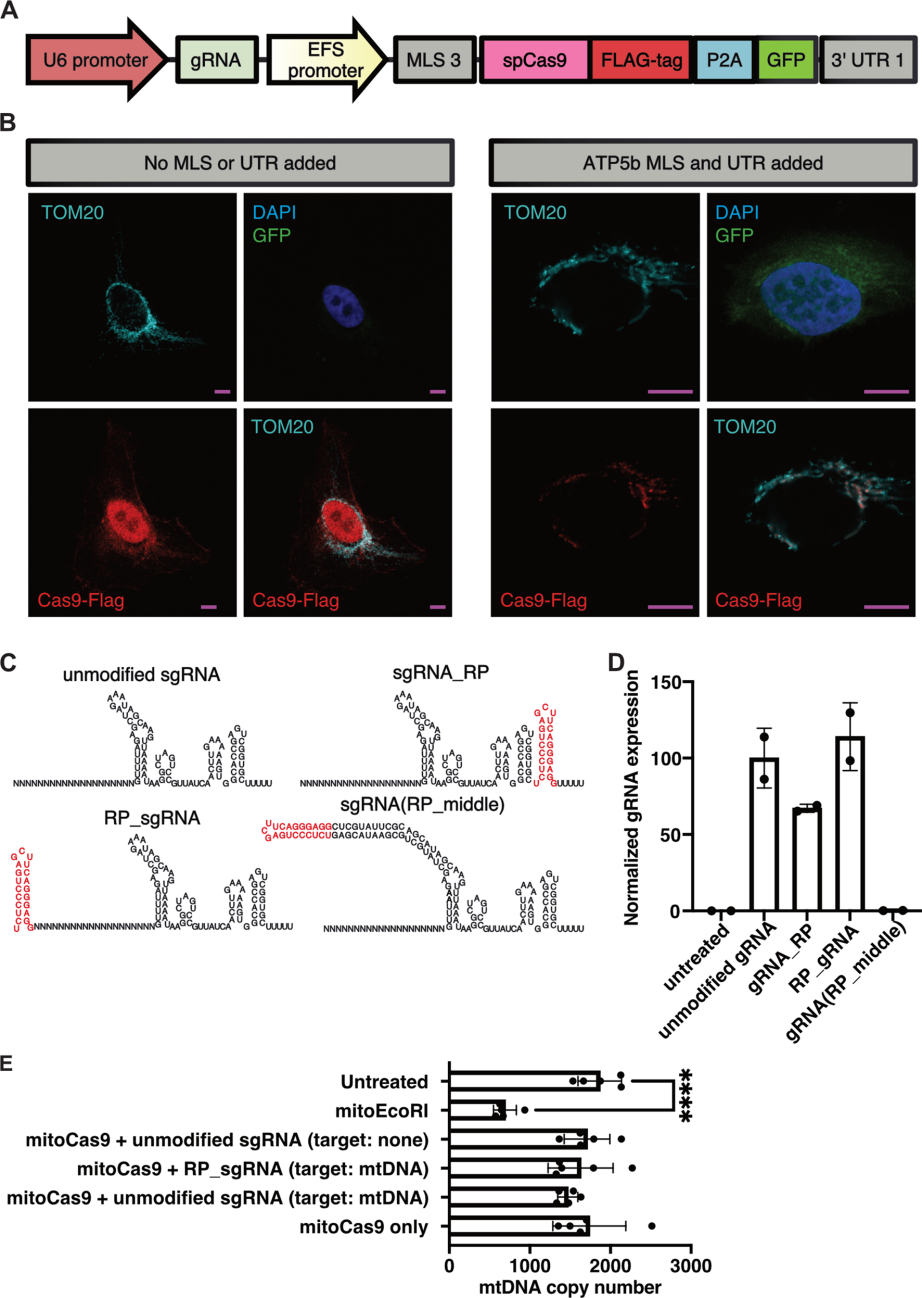
Delivery of spCas9 into mitochondria. (**A**) MLS3 (ATP5b) and UTR1 (ATP5b) were attached to FLAG-tagged spCas9 to facilitate delivery into mitochondria. GFP, separated from spCas9 by a P2A sequence, was included in the construct to enable identification of successfully transfected cells. (**B**) Representative confocal microscopy images of HT1080 cells transfected with spCas9 constructs with or without MLS3 and UTR1 attached. Mitochondria are visualized by TOM20 staining (cyan). Scale bars: 10 μm. (**C**) RP RNA loop sequence (red) was added to different positions of the gRNA to facilitate gRNA delivery to mitochondria. (**D**) Relative expression levels of different sgRNA combinations in HEK 293T cells 2 days post-transfection (n = 2). (**E**) mtDNA copy numbers in HEK293 T cells transfected with different constructs, 2 days post-transfection. Only successfully transfected, GFP + cells were sorted and analyzed with qPCR (n = 5, ****p < 0.0001).

Before starting to target Cas9 to mitochondria, we first attached several MLS/3’-UTR combinations (Fig. 1A, Supplementary Table 1) to GFP, and assessed co-localization of the expressed GFP protein and mitochondria with confocal microscopy imaging. We found that most of the tested MLS/3’-UTR combinations were highly efficient at targeting GFP to mitochondria, as detected by colocalization of GFP and a mitochondria-staining dye in HEK 293T cells (Fig. 1B). No GFP expression outside of mitochondria was detected, except for MLS4/UTR4. We chose combination MLS3/UTR1 and attached it, together with a FLAG-tag, to Cas9 (Fig. 2A). HT1080 cells were then transfected with Cas9-encoding plasmids. After fixation and antibody staining, confocal microscopy revealed that Cas9 with MLS3/UTR1 is exclusively localized to mitochondria. In contrast, Cas9 without any localization sequence was distributed throughout the cytoplasm and nucleus (Fig. 2B).

Next, we set out to design a gRNA that can be targeted to mitochondria in order to enable site-specific Cas9 cutting. We modified gRNA by attaching an RP-loop sequence which had previously been reported to facilitate both mRNA and tRNA import to mitochondria^23,24^. Since it was unclear how the RP-loop would impact gRNA expression and function, we inserted the sequence at three different positions of the gRNA (Fig. 2C). gRNA expression was driven by a U6 promoter that was present on the same plasmid as Cas9. We performed RT-qPCR to measure the expression level of each modified gRNA in transfected HEK 293T cells. Addition of the RP-loop to the 5’- or 3’-end did not impair gRNA expression. Insertion of the loop to the middle of the sgRNA led to a complete loss of its expression, and we excluded this configuration from further analysis (Fig. 2D).

Confirming localization of a small RNA to the mitochondrial matrix is highly challenging, and most assays are plagued by false-positive results, which can be caused by RNA that is attached to the outside of mitochondria or mitoplasts. To avoid having to work with unreliable small RNA localization assays, we decided to directly assess whether the modified gRNAs could successfully facilitate Cas9-mediated degradation of wild-type mtDNA. Since linearized mtDNA is rapidly degraded, successful cutting of mtDNA would lead to a decrease in mtDNA copy number, which can be detected with qPCR. Since we already achieved targeting the Cas9 protein to mitochondria, we expected that successful targeting of gRNA to the mitochondria would lead to quantifiable mtDNA depletion. For the quantification of mtDNA copy number, we adapted a previously described multiplexed qPCR assay^25^ to compare mtDNA levels in each condition relative to the nuclear DNA encoded gene B2M. We also quantified the effect of the different constructs on mitochondrial gene expression with RT-qPCR. HEK 293T cells were transfected with the different constructs that encoded mitochondria-targeted Cas9 and different gRNA configurations. The gRNAs were designed to target the mitochondrial origin of replication OriH (mtORI, position 16,482–16,501). The non-targeting control gRNA does not target any sequence within the human genome. Successfully transfected, GFP+ (see Fig. 2A) cells were sorted after 2 days in culture. DNA or RNA was extracted immediately after the sort. As a positive control for successful mtDNA cutting, we cloned the same MLS3/UTR1 combination onto the restriction enzyme EcoRI, which has three target sites within human mtDNA.

**Figure 3:**
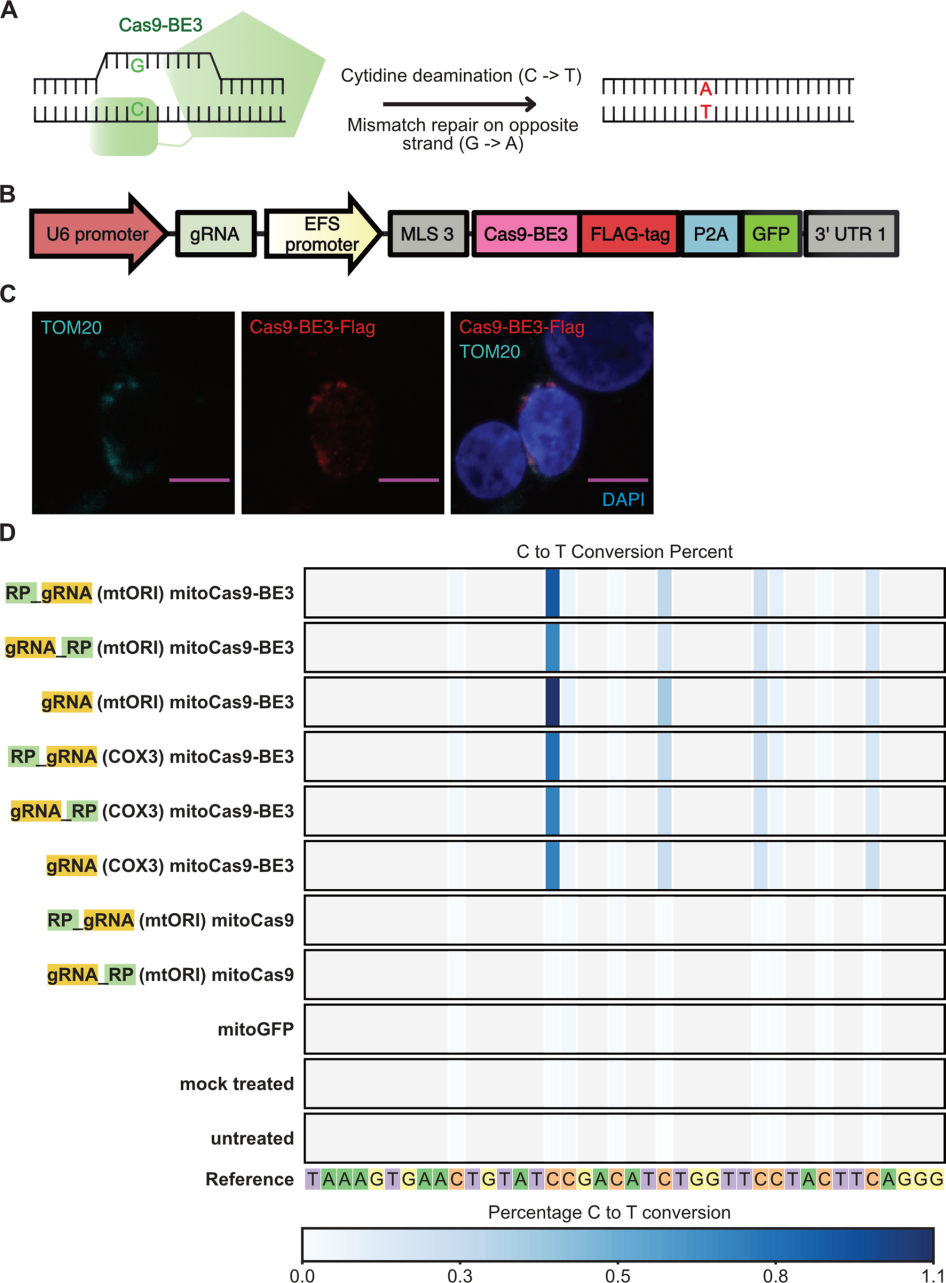
Delivery of base editor Cas9-BE3 into mitochondria. (**A**) Schematic outline of Cas9-BE3 activity. (**B**) Outline of mitochondria-targeted Cas9-BE3. (**C**) Confocal microscopy showing colocalization of FLAG-tagged Cas9-BE3 with mitochondria in HEK 293T cells. Scale bars: 10 μm. (**D**) Frequency of C to T conversions in HEK 293T cells treated with different constructs. Selected part of site within mtDNA that was targeted by mtORI gRNAs is shown. Only successfully transfected, GFP + cells were sorted and analyzed with amplicon next generation sequencing.

Our qPCR analysis revealed that mitochondria-targeted restriction enzyme EcoRI strongly reduces mtDNA levels by over 60% from approximately 1870 to 690 mtDNA copies per cell. None of the tested Cas9/sgRNA combinations led to a significant reduction of mtDNA levels in HEK 293T cells (Fig. 2E). Similarly, mitochondria-targed EcoRI slightly reduced the expression levels of the mitochondrial transcript of MT-CO3, while none of the tested Cas9 constructs had a detectable effect (Supplementary Fig. 1A). There were several possible reasons for this. One possibility was that Cas9 activity in mitochondria might not be strong enough to cause a detectable effect on mtDNA copy number. Unlike canonical restriction enzymes such as EcoRI, spCas9 is a single-turnover enzyme^26^. This means that a single Cas9 molecule cannot cleave more than a single mtDNA molecule. This limited rate of Cas9 cleavage activity may have been lower than the rate at which mtDNA is replicated and renewed. A more likely possibility for our failure to detect any meaningful mtDNA depletion is that our approach for targeting the gRNA for mitochondrial import was not successful. One general problem with this assay for detecting mtDNA cutting is the high variability of mtDNA copy numbers between biological replicates. Even in the untreated condition, where an identical starting cell population was separated into 5 different wells prior to a 2 day culture, mtDNA levels varied by 600 average copies per cell (> 30% variation). Previously published research showed that mtDNA levels in individual cells of the same cell line are highly variable and range from a few hundred to more than 20,000 copies^27^. While using qPCR-based mtDNA copy number detection as an assay is suitable for detecting extreme effects on mtDNA levels, such as those caused by EcoRI-mediated DNA cleavage, it is not sensitive enough to distinguish more subtle effects from the background noise of biological variability. Therefore, we decided to use a different approach which would lead to an easily detectable and unambiguous signal. To this end, we chose to use the recently developed C to T base editors, which consist of a Cas9-nickase fused to a cytidine deaminase and uracil DNA glycosylase inhibitor^28^. Unlike regular Cas9, they do not cause double-strand breaks, but install a precise C to T change at the targeted site. Such base editing events are easily detectable with next generation sequencing (NGS), even if they happen at a low rate (Fig. 3A). We hypothesized that this system would drastically reduce the issues with biological variability that confounded our previous attempts. Furthermore, the establishment of a tool to install precise C to T edits in mtDNA could be helpful for the development of gene therapies of monogenic mitochondrial diseases caused by T to C mutations. It would also enable the generation of disease models by installing point mutations at defined sites within the mtDNA, and the study of the resulting phenotype. To see if this is possible, we first attached the previously described MLS and UTR to Cas9-BE3 (Fig. 3B), and confirmed that the protein was successfully imported to mitochondria of HEK 293T cells with confocal microscopy (Fig. 3C). This construct also contained GFP to enable identification and sorting of transfected cells. We then selected two sgRNAs that target distinct sites; the mitochondrial origin of replication (mtORI), and COX3 within the mitochondrial DNA, with no potential off-target sites in the nuclear DNA (Supplementary Fig. 2). With each of these sgRNA designs, we then created constructs with either unmodified sgRNA backbone, or with the previously described mitochondria-targeting RP sequence attached to the 5’ or 3’ end. We transfected HEK 293T cells with these different constructs and extracted DNA from successfully transfected (GFP+) cells after 4 days in culture. The complete list of constructs used in this experiment is shown in Fig. 3D. To detect mtDNA editing in these samples, we then amplified fragments that covered the targeted sites and performed amplicon sequencing to measure the rate of C to T conversions. We detected low levels of C to T conversion (~ 1%) at the mtORI site in all 6 samples that were treated with mitochondria-targeted Cas9-BE3, regardless of which sgRNA was used (Fig. 3D, Supplementary Fig. 3A and 4). None of the 5 distinct control samples which did not contain a base editor (see Fig. 3D for complete list) had any detectable rate of C to T conversions. This strongly indicates that the detected C to T conversion were in fact caused by mitochondria-targeted Cas9-BE3. Surprisingly, C to T conversions at the mtORI site were detected both with mtORI-targeting, and with COX3-targeting sgRNAs. The most likely explanation for this observation is that the detected base-editing activity was caused by low levels of nonspecific, gRNA-independent off-target activity of Cas9-BE3. Nonspecific base editing activity by Cas9-BE3 has previously been observed on nuclear DNA as well^29,30^. Interestingly, no elevated levels of C to T conversions were detected at the COX3 site (Supplementary Fig. 3B) in any samples. The mtORI site might be more susceptible to unspecific base editing activity because it naturally occurs in a D-loop configuration which exposes single-stranded DNA, which is the preferred substrate for base editors. Taken together, these results demonstrate that Cas9-BE3 itself can function within mitochondria and modify mtDNA, but also that our attempts to deliver sgRNA to mitochondria were not successful.

There have been several reports that describe gRNA import and Cas9 activity in mitochondria^24,31–34^, but the results and conclusions from such studies have been controversial and challenged by other groups^35–38^. Our results strongly suggest that adding an RP sequence to gRNA is not a viable approach for facilitating gRNA import. A potential limitation of this study is that the used gRNAs have not been confirmed to be able to facilitate DNA cutting or base editing. It is, however, unlikely that both tested gRNAs have less than 1% efficiency in the base editing experiment. Additionally, more than 8 years after the CRISPR Cas9 system has been described and utilized, no convincing reports of using Cas9 for the efficient modification of mtDNA have been released. Taken together, our results and the fact that there are still no convincing reports of Cas9-based modification of mtDNA strongly suggest that there is no RNA import pathway that can be exploited to channel RNA into mammalian mitochondria. There have, however, been reports of nucleic acid delivery into yeast mitochondria by physical transfection methods such as a gene gun^39^. It remains to be explored if similar methods could also be used to target mammalian mitochondria. The highly sensitive Cas9-BE3-based assay could be scaled up to perform large-scale screens of different modified gRNAs and delivery methods, and this will be a valuable tool for researchers who want to explore this further. While Cas9-BE3 has allowed us to confirm that mitochondria-targeted cytosine base editors can be active in mitochondria, future studies should use more recent base editors with improved editing efficiencies^40,41^. In 2020, Mok et al*.*^42,43^ developed a TALEN-guided base editor, which enables the creation of site-specific modifications of mtDNA. Despite this, extending the benefits that Cas9 has provided in the context of nuclear DNA to mtDNA would still be a major breakthrough. Until the significant challenges associated with gRNA import are overcome, Cas9-based mtDNA modifications will continue to remain out of reach.

## Methods

### Cell culture

HEK 293T cells or HT-1080 cells (DMSZ, German Collection of Microorganisms and Cell Cultures) were cultured in DMEM medium (high glucose, pyruvate, Cytiva) with 10% FCS and 1% penicillin–streptomycin. Medium for cells that were transfected with constructs that might reduce mtDNA copy numbers was supplemented with uridine (50 μg/mL, U3003, Sigma-Aldrich) to prevent toxicity from mtDNA loss, as previously described^44,45^. Cells were cultured at 37 °C and 5% CO_2_. Plasmids were delivered to cells with the calcium phosphate transfection method^46^. 1.9 μg plasmid per well was used for 70% confluent cells in 24-well plates.

### Molecular cloning

All constructs were created by modifying the pLCv2 vector (Addgene #52961,^47^) using standard molecular biology techniques. The constructs containing mitochondria-targeted GFP were created by amplifying the MLS and 3’ UTR sequences selected based on previous publications^9,20,44,48,49^ and described in Supplementary Table 1 from human cDNA and attaching them to GFP. In all constructs with mitochondria-targeted proteins, a SAGGGGS linker was placed between the MLS and the protein. Constructs containing mitochondria-targeted Cas9 were created by removing the usually present nuclear localization sequence (NLS), and by attaching MLS3 (ATP5b) and UTR1 (ATP5b). sgRNA expression was driven by a U6 promoter, and for certain sgRNAs, the RP sequence^23^ TCTCCCTGAGCTTCAGGGAGG was attached at different positions as shown in Fig. 2C. For the experiment in Fig. 2D sgRNAs targeted GCCGTAGATGCCGTCGGAAA (COX3, mtDNA), and in Fig. 2E sgRNA targeted AGTGAACTGTATCCGACATC (mtORI, mtDNA) and GGCCCAACATCCTCGTGTCCA (negative control, no target). Mitochondria-targeted EcoRI was created in a way that was inspired by a publication from Kukat et al.^45^. In brief, EcoRI was amplified from pAN4 plasmid^50^, and cloned between the previously described MLS3 and UTR1. Cas9-BE3 from Addgene plasmid #73021^28^ was also cloned between MLS3 and UTR1, together with the previously mentioned mtORI and COX3-targeting sgRNAs.

### Confocal microscopy

Cells were cultured on poly-D-lysine coated glass slides and fixed with methanol and 4% formaldehyde solution. If Mitotracker CMXRos (M-7512, Thermo Fisher Scientific) was used, it was added before fixation according to the manufacturer’s instructions. Fixed cells were washed and blocked for 1 h with 1% goat serum in PBS, and stained with primary antibodies rabbit anti-Tom20 (1:500, FL-145, Santa Cruz Biotechnology) and mouse anti-FLAG (1:200, F1804, Sigma-Aldrich) in a humidified chamber at 4 °C overnight. Cells were washed again and stained with secondary antibodies goat anti-rabbit Cy5 (1:200, ab97077, Abcam), and goat anti-mouse Alexa Fluor 568 (1:500, ab175701, Abcam) for 1.5 h. After additional washing steps and staining with DAPI, cells were imaged using a Zeiss LSM 780 confocal microscope.

### Flow cytometry

GFP-positive cells were sorted into fresh medium using a FACSAria II or FACSAria III Cell Sorter (BD Biosciences). 7AAD staining (1:200) was used to exclude dead cells.

### Validation of gRNA expression by RT-qPCR

Total RNA of HEK 293T cells that were transfected with constructs containing different sgRNA configurations was extracted 2 days post transfection with standard TRIzol RNA isolation followed by DNase treatment. RNA was reverse-transcribed with Superscript III reverse transcriptase, using random hexamer primers. SYBR green-based qPCR (7900HT Fast Real-Time PCR System (Applied Biosystems)) was performed using GAPDH as a reference gene (Primers GAPDH: TGCACCACCAACTGCTTAGC, and GGCATGGACTGTGGTCATGAG) and primers specific for all tested sgRNA configurations (Primers sgRNAs: GCCGTAGATGCCGTCGGAAAG, and CGACTCGGTGCCACTTTTTCAAGTTG).

### mtDNA copy number and gene expression determination with qPCR and RT-qPCR

The same batch of HEK 293T cells was split into 5 separate wells per condition and transfected independently. After 2 days, 100,000 to 200,000 successfully transfected GFP-positive cells per replicate were sorted, and DNA was extracted using the QIAamp DNA Blood Mini Kit (Qiagen). The DNA concentration of all samples and replicates was determined and then adjusted by dilution with H_2_O, such that all samples had the same concentration. A multiplexed qPCR assay based on previously published work^25^ was performed using a probe targeting the nuclear gene B2M (VIC) and a probe targeting minor arc of mtDNA (6FAM), with the TaqMan Gene Expression Master Mix (#4369016, Applied Biosystems), and the 7900HT Fast Real-Time PCR System. The primers targeting mtDNA were CTAAATAGCCCACACGTTCCC and AGAGCTCCCGTGAGTGGTTA. The primers targeting B2M were GCTGGGTAGCTCTAAACAATGTATTCA and CCATGTACTAACAAATGTCTAAAATGGT. The qPCR probes used to detect mtDNA and B2M were 6FAM-CATCACGATGGATCACAGGT(NFQ), and VIC-CAGCAGCCTATTCTGC(NFQ), respectively. Based on the Ct values obtained from the qPCR reaction, the relative ratio of mtDNA to nuclear DNA was determined. The mtDNA to nuclear DNA ratio reflects the average mtDNA copy number of each analyzed sample.

In another experiment with the same setup, RNA was extracted from sorted GFP-positive cells using the RNeasy Micro Kit (Qiagen) and reversed transcribed with the SuperScript III First-Strand Synthesis System (Thermo Fisher Scientific). RT-qPCR was performed using the TaqMan Gene Expression Master Mix (#4369016, Applied Biosystems), and TaqMan probes (Hs02596866_g1, Hs02800695_m1) targeting the mitochondrial gene MT-CO3 and the reference gene HPRT.

### Next generation sequencing

Genomic DNA from 293T cells was extracted 4 days post transfection and used for primary amplification of the mtORI and COX3 sites. Primary amplification using Phusion HS Polymerase Master Mix (Thermo Fisher Scientific F531S), 30 amplification cycles, 2 ng of input genomic DNA (analyzed by Qubit; Themo Fisher Scientific Q33240), was conducted according to manufacturer-recommended conditions. The mtORI amplicon primers including i7/i5 adapters were GTCTCGTGGGCTCGGAGATGTGTATAAGAGACAGTCTCCTCGCTCCGGGCCCAT and TCGTCGGCAGCGTCAGATGTGTATAAGAGACAGGGGGAA CGTGTGGGCTATTTAGGCT.

COX3 amplicon primers including i7/i5 adapters were GTCTCGTGGGCTC GGAGATGTGTATAAGAGACAGTCAATCACCTGAGCTCACCATAGTC and TCGTCGGCAGCGTCAGATGTGTATAAGAGACAGCCGTGGAAGCCTGTGGCTA. The primary PCR products were purified using AmpureXP beads (Beckman Coulter A63880) with manufacturer-recommended protocol (1.8 × bead ratio) and measured for concentration using Qubit. Next, primary PCR products were diluted and samples of 2 ng of input DNA were indexed using i5/i7 Illumina indexing primers (Nextera XT Index Kit v2 Set A, FC-131-2001) over 12 cycles of amplification, using Phusion HF Polymerase Master Mix. Indexed libraries were then diluted and pooled according to Illumina-recommended protocol for sequencing on Illumina NextSeq machine (300 cycles paired end). Amplicon sequencing data was analyzed using CRISPResso2^51^ using the base editor output option, with paired end reads, –min_average_read_quality 28, and –min_single_bp_quality 13.

### Statistics

When comparing multiple groups, GraphPad Prism 8 was used to perform One-Way analysis of variance (ANOVA) with Tukey’s multiple comparison test. Data is shown as Mean ± SD and significance is indicated with asterisks (* < 0.05, **** *p* < 0.0001).

## Supplementary Information

Supplementary Information 1.

Supplementary Information 2.

## Data Availability

The next generation sequencing dataset is available in the Sequence Read Archive (NCBI) and is accessible under the Bioproject ID PRJNA851606. Reviewer link: https://dataview.ncbi.nlm.nih.gov/object/PRJNA851606.
