# Supplementary material for: Site-specific CRISPR-based mitochondrial DNA manipulation is limited by gRNA import
Source: Sci Rep. 2022 Nov 4;12:18687. doi: 10.1038/s41598-022-21794-0 (PMC9636205; doi:10.1038/s41598-022-21794-0)
Supplement: Supplementary file 1 — Supplementary Information 1. [file 41598_2022_21794_MOESM1_ESM.pdf]

Supplementary Information

Site-specific CRISPR-based mitochondrial DNA manipulation is limited by gRNA import

Ludwig Schmiderer[1,#], David Yudovich[1], Leal Oburoglu[1], Martin Hjort[2,3], Jonas Larsson[1,#]

[1] Division of Molecular Medicine and Gene Therapy, Department of Laboratory Medicine and Lund Stem Cell Center, BMC A12, Lund University, 221 00 Lund, Sweden

[2] Chemical Biology and Therapeutics, Department of Experimental Medical Science, Lund University, 221 00 Lund, Sweden

[3] Navan Technologies, MBC Biolabs, San Carlos, CA 94070, USA

[#] Co-corresponding authors

Lead author: Ludwig Schmiderer

Email: ludwig.schmiderer@med.lu.se

Address of Lead Author

Ludwig Schmiderer, BMC A12, 221 84 Lund, Sweden. Phone +46 46 2220580

Supplementary Figure legends

Supplementary Figure 1. mRNA levels of mitochondrial gene MT-CO3 relative to the reference gene HPRT in HEK 293T cells transfected with different constructs, 2 days post transfection. Only successfully transfected, GFP+ cells were sorted and analyzed with RT-qPCR ($n = 5$, * $p < 0.05$).

Supplementary Figure 2. Illustration of human mtDNA. The positions of gRNAs targeting mtORI and COX3 are indicated. Image was generated with SnapGene.

Supplementary Figure 3. Extended view (complete amplicon) of C to T conversions in HEK 293T cells transfected with different constructs. (A) shows the mtORI site, and (B) the COX3 site.

Supplementary Figure 4. Total frequency of all possible substitutions in HEK 293T cells transfected with different constructs. A low background level of substitutions of all kinds is present in all samples, but only samples with mitochondria-targeted Cas9-BE3 show increased levels of C to T substitutions.

Supplementary Figure 1

A

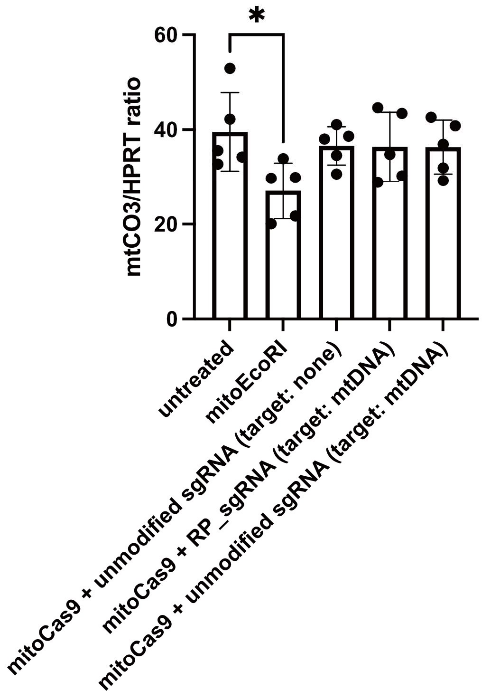

Supplementary Figure 1. mRNA levels of mitochondrial gene MT-CO3 relative to the reference gene HPRT in HEK 293T cells transfected with different constructs, 2 days post transfection. Only successfully transfected, GFP+ cells were sorted and analyzed with RT-qPCR (n = 5, * $p < 0.05$).

Supplementary Figure 2

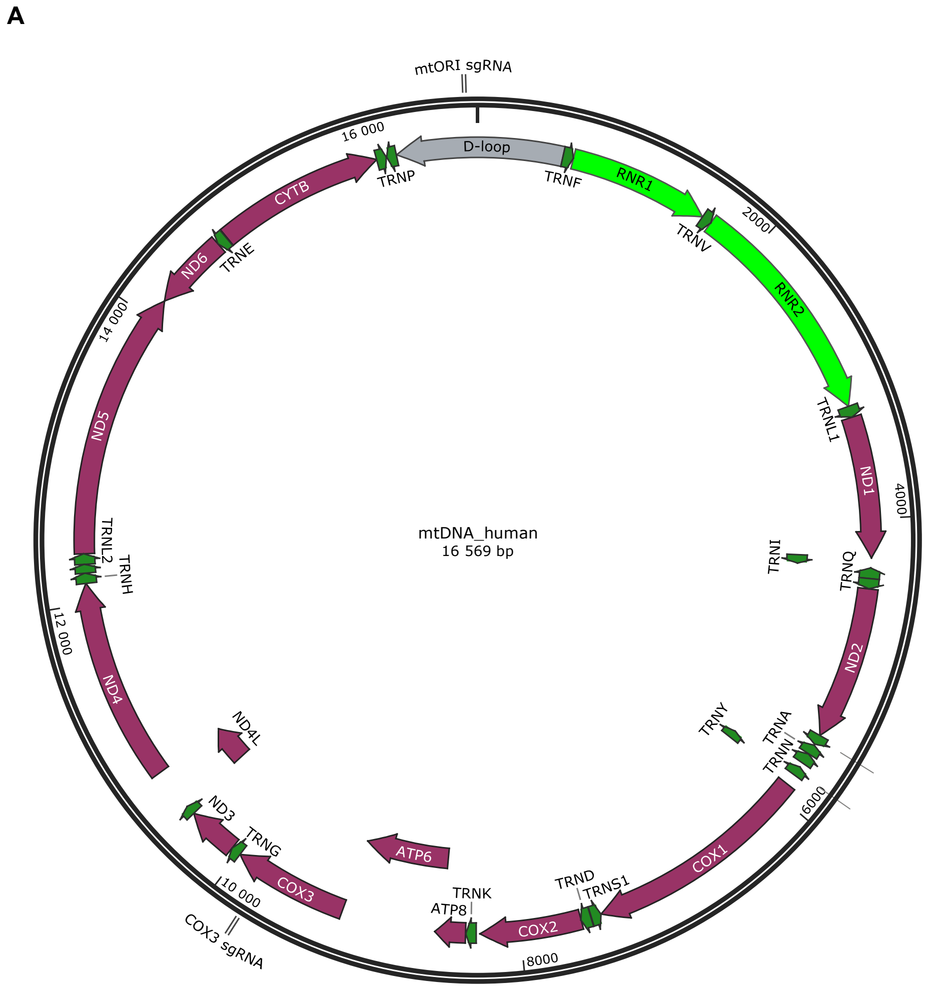

Supplementary Figure 2. Illustration of human mtDNA. The positions of gRNAs targeting mtORI and COX3 are indicated. Image was generated with SnapGene.

Supplementary Figure 3

A

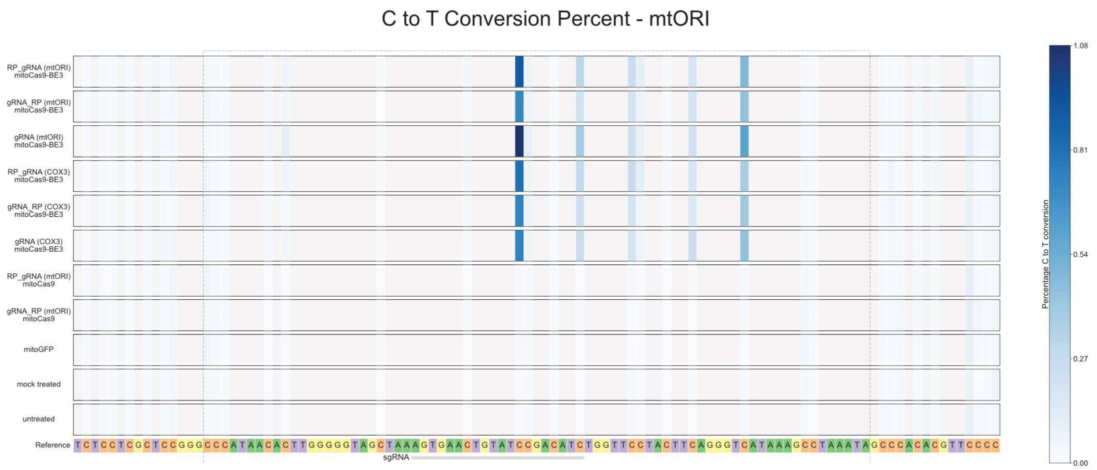

B

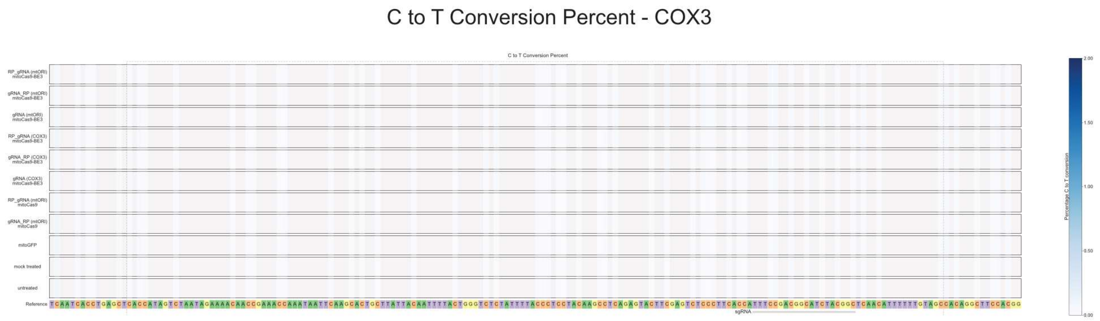

Supplementary Figure 3. Extended view (complete amplicon) of C to T conversions in HEK 293T cells transfected with different constructs. (A) shows the mtORI site, and (B) the COX3 site.

Supplementary Figure 4

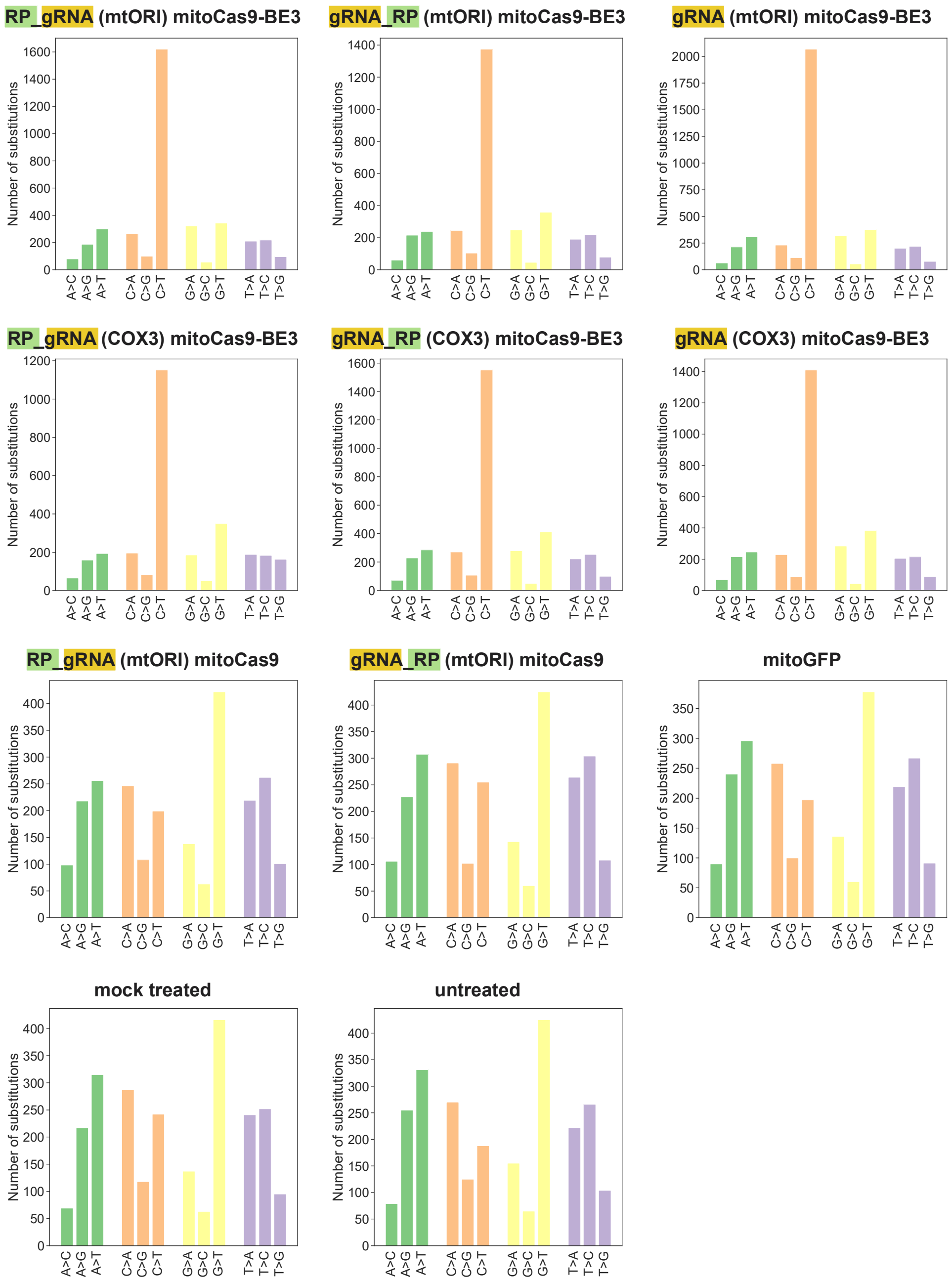

Supplementary Figure 4. Total frequency of all possible substitutions in HEK 293T cells transfected with different constructs. A low background level of substitutions of all kinds is present in all samples, but only samples with mitochondria-targeted Cas9-BE3 show increased levels of C to T substitutions.
