# Supplementary material for: Site-specific CRISPR-based mitochondrial DNA manipulation is limited by gRNA import
Source: Sci Rep. 2022 Nov 4;12:18687. doi: 10.1038/s41598-022-21794-0 (PMC9636205; doi:10.1038/s41598-022-21794-0)
Supplement: Supplementary file 2 — Supplementary Information 2. [file 41598_2022_21794_MOESM2_ESM.pdf]

Supplementary Table 1. List of mitochondrial localization sequences (MLS) and 3' UTRs used in this study. All sequences originate from *Homo sapiens* genes.

	Gene	Protein or DNA sequence used in constructs
MLS1	SOD2	MLSRAVCGTSRQLAPVLGYLGSRQKHSLPD
MLS2	COX8A	MSVLTPLLLRGLTGSARRLPVPRAKIHSL
MLS3	ATP5b	MLGFVGRVAAAPASGALRRLTPSASLPPAQLLLRAAPTAV<br>HPVRDYAAQ
MLS4	COX10	MAASPHTLSSRLLTGCVGGSVWYLERRT
UTR1	ATP5b	GGGGTCTTTGTCCTCTGTACTGTCTCTCTCCTTGCCCCTA<br>ACCCAAAAAGCTTCATTTTTCTGTGTAGGCTGCACAAG<br>AGCCTTGATTGAAGATATATTCTTTCTGAACAGTATTTA<br>AGGTTTCCAATAAAATGTACACCCCTCAGAA
UTR2	SOD2 variant 1	ACCACGATCGTTATGCTGAGTATGTTAAGCTCTTTATGA<br>CTGTTTTTGTAGTGGTATAGAGTACTGCAGAATACAGT<br>AAGCTGCTCTATTGTAGCATTTCTTGATGTTGCTTAGTC<br>ACTTATTTCATAAACAACTTAATGTTCTGAATAATTTCTT<br>ACTAAACATTTTGTTATTGGGCAAGTGATTGAAAATAG<br>TAAATGCTTTGTGTGATTGA
UTR3	SOD2 variant 2	ACCACGATCGTTATGCTGATCATACCCTAATGATCCCAG<br>CAAGATAATGTCCTGTCTTCTAAGATGTGCATCAAGCCT<br>GGTACATACTGAAAACCCTATAAGGTCCTGGATAATTT<br>TTGTTTGATTATTCATTGAAGAAACATTTATTTTCCAATT<br>GTGTGAAGTTTTTGACTGTTAATAAAAGAATCTGTCAA<br>CCATCAA
UTR4	COX10	GAGCACTGGGACGCCCACCGCCCCTTTCCCTCCGCTGC<br>CAGGCGAGCATGTTGTGGTAATTCTGGAACACAAGAA<br>GAGAAATTGCTGGGTTTAGAACAAGATTATAAACGAAT<br>TCGGTGCTCAGTGATCACTTGACAGTTTTTTTTTTTTTTA<br>AATATTACCCAAAATGCTCCCCAAATAAGAAATGCATC<br>AGCTCAGTCAGTGAATACAAAAAAGGAATTATTTTTCC<br>CTTTGAGGGTCTTTATACATCTCTCCTCCAACCCCACCCT<br>CTATTCTGTTTCTTCCTCCTCACATGGGGGTACACATAC<br>ACAGCTTCCTCTTTTGGTTCCATCCTTACCACCACACCAC<br>ACGCACACTCCACATGCCCAGCAGAGTGGCACTTGGTG<br>GCCAGAAAGTGTGAGCCTCATGATCTGCTGTCTGTAGT<br>TCTGTGAGCTCAGGTCCCTCAAAGGCCTCGGAGCACCC<br>CCTTCCTGGTGACTGAGCCAGGGCCTGCATTTTTGGTTT<br>TCCCCACCCCACACATTCTCAACCATAGTCCTTCTAACA<br>ATACCAATAGCTAGGACCCGGCTGCTGTGCACTGGGAC<br>TGGGGATTCCACATGTTTGCCTTGG
